# Open versus arthroscopic ankle arthrodesis: a systematic review and meta-analysis

**DOI:** 10.1186/s13018-020-01708-4

**Published:** 2020-05-24

**Authors:** Tsz Ngai Mok, Qiyu He, Soundarya Panneerselavam, Huajun Wang, Huige Hou, Xiaofei Zheng, Jinghua Pan, Jieruo Li

**Affiliations:** 1 Department of Sport Medicine, First Affiliated Hospital of Jinan University, Guangzhou, 510630 Guangdong China; 2 International School, Jinan University, Guangzhou, 510632 Guangdong China; 3 Department of Gastrointestinal Surgery, First Affiliated Hospital of Jinan University, Guangzhou, 510630 Guangdong China

**Keywords:** Arthrodesis, Ankle Joint, Arthroscopy, Open surgery

## Abstract

**Background:**

Osteoarthritis (OA) is a growing health concern that affects approximately 27 million people in the USA and is associated with a $185 billion annual cost burden. Choosing between open surgery and arthroscopic arthrodesis for ankle arthritis is still controversial. This study compared arthroscopic arthrodesis and open surgery by performing a systematic review and meta-analysis.

**Methods:**

For the systematic review, a literature search was conducted in 4 English databases (PubMed, Embase, Medline and the Cochrane Library) from inception to February 2020. Three prospective cohort studies and 7 retrospective cohort studies, enrolling a total of 507 patients with ankle arthritis, were included.

**Results:**

For fusion rate, the pooled data showed a significantly higher rate of fusion during arthroscopic arthrodesis compared with open surgery (odds ratio 0.25, 95% CI 0.11 to 0.57, *p* = 0.0010). Regarding estimated blood loss, the pooled data showed significantly less blood loss during arthroscopic arthrodesis compared with open surgery (WMD 52.04, 95% CI 14.14 to 89.94, *p* = 0.007). For tourniquet time, the pooled data showed a shorter tourniquet time during arthroscopic arthrodesis compared with open surgery (WMD 22.68, 95% CI 1.92 to 43.43, *p* = 0.03). For length of hospital stay, the pooled data showed less hospitalisation time for patients undergoing arthroscopic arthrodesis compared with open surgery (WMD 1.62, 95% CI 0.97 to 2.26, *p* < 0.00001). The pooled data showed better recovery for the patients who underwent arthroscopic arthrodesis compared with open surgery at 1 year (WMD 14.73, 95% CI 6.66 to 22.80, *p* = 0.0003).

**Conclusion:**

In conclusion, arthroscopic arthrodesis was associated with a higher fusion rate, smaller estimated blood loss, shorter tourniquet time, and shorter length of hospitalisation than open surgery.

## Background

Osteoarthritis (OA) is a growing health concern that affects approximately 27 million people in the USA and is associated with a $185 billion annual cost burden [1]. Disabling or even debilitating functional impairment is the main symptom of end-stage ankle arthritis [2]. It can drastically alter the quality of life of a patient. Arthrodesis is one of the last options for patients when conservative treatment fails [3, 4].

Arthrodesis, via open surgery, is the traditional option for treating ankle arthritis, chronic instability, and degenerative deformity [5]. Pain relief and functional improvement of a foot with ankle degeneration are reasons why ankle arthrodesis might be highly recommended as a reliable treatment. However, arthrodesis alters biochemical performance and may cause foot pain, joint arthritis, and bone fracture [6–8]. In the last decade, arthroscopic arthrodesis, which is an advanced technique for treating ankle problems, has been used. It has been an available option since 1983 [9]. It has been reported that arthroscopic arthrodesis is less invasive, and patients suffer less pain after surgery. However, the presence of poor skin and poor vascularity are relative contraindications [10]. The contraindications must be aware as they can lead to severe misalignments and bone loss [11].

Choosing between open surgery and arthroscopic arthrodesis for ankle arthritis is still controversial [12]. However, to our knowledge, only one systematic review and meta-analysis comparing the outcomes of open and arthroscopic methods of ankle fusion is available in the literature, but it did not contain data on postoperative improvement [3]. This study evaluated the fusion rate, effectiveness, complications and operative improvements by performing a systematic and meta-analysis that focused on all studies that met our criteria: studies that were comparing arthroscopic arthrodesis and open surgery for patients with ankle arthritis.

## Methods

### Search strategy

**Fig. 1:**
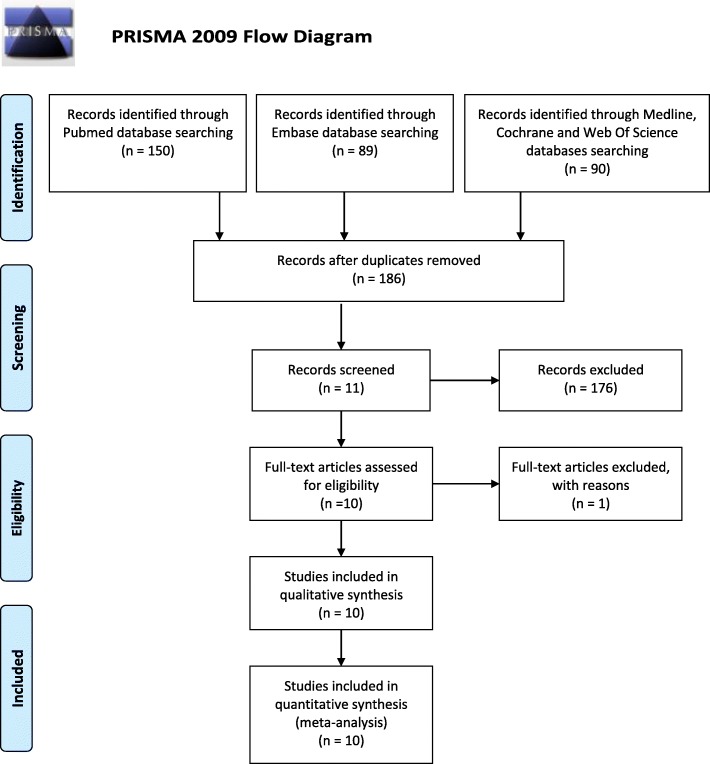
PRISMA 2009 flow diagram

For the systematic review, a literature search was conducted using 4 English databases (PubMed, Embase, Medline and the Cochrane Library) from inception to February 2020. To maintain high sensitivity in our research, we decided to include relevant medical subject heading (MeSH) terms, common keywords, and a comprehensive combination. No language restriction and no filters were set for the strategy. All the relevant published article bibliographies were reviewed. A total of 142 references were removed due to duplication, and 186 references were imported for initial screening of titles and abstracts (Fig. 1).

### Inclusion and exclusion criteria

Two examiners screened all the references, including the titles and abstracts, separately so that the eligibility criteria could be achieved. Irrelevant articles and non-full-text references were excluded. The inclusion criteria were as follows: (1) patients with arthritis, including post-traumatic arthritis, osteoarthritis, and end-stage arthritis, or patients with ankle instability; (2) comparative studies of open arthrodesis surgery and arthroscopic arthrodesis; and (3) one or more outcomes of interest shown in the studies. The excluded studies were (1) not original articles or (2) preclinical studies.

### Data extraction and quality assessment

The following variables were extracted and double-checked by the reviewers independently, and a quality assessment was performed. The Newcastle–Ottawa Scale (NOS) was used to assess the risk of bias of the observational studies. There is a maximum of nine stars in 3 domains (8 items): the selection of the study groups, the compatibility of the groups, and the ascertainment of the outcome of interest [13]. According to the scale, there are only low risk and high risk. Low risk was rated as one star, and high risk was rated as no star. Nine stars in a study indicated a low risk of bias, seven to eight stars in a review indicated a moderate risk of bias, and six or fewer stars in a study indicated a high risk of bias. Furthermore, a methodological index for non-randomised studies (MINORS) was also used for the assessment. Twelve items were included in the checklist: clearly stated aim, inclusion of consecutive patients, prospective data collection, endpoints appropriate to study aim, follow-up period appropriate to study aim, less than 5% lost to follow-up, prospective calculation of study size, an adequate control group, contemporaneous groups, baseline equivalence of the groups and adequate statistical analyses. A score of 0–2 is given for each item. The first 8 items are for those without a control group, and the maximum score is 16. The last 4 items and the previous items are for those with a control group, and the maximum score is 24. Zero indicated no record; 1 indicated an unclear recording; and 2 indicated that the outcome was fully reported in the article. Besides, the Coleman methodology score was added in order to indicate that a given study generally minimizes confounding factors and biases [14]. It is separated (Part A and Part B). The total score of the methodology is 100. Two reviewers discussed whether there was any disagreement that needed to be resolved [15]. A third author was consulted when no agreement could be achieved. The baseline demographic and clinical characteristics of the study participants, including age, sex ratio, body mass index (BMI), sample size and lesion types, were recorded. At least 15 surgeons from different institutes participated in both the arthroscopic and open surgeries. The minimum duration of data collection was three years, and Peterson et al. [16] reported a maximum duration of 11 years (Table 1). Regarding the information about the interventions, the information collected about open and arthroscopic arthrodesis was included in the ratio in each study. In the investigation, we defined clinical fusion as a stable, painless ankle [16]. Adverse events and postoperative complications, the overall complication rate and the infection rates were recorded. Operation time, estimated blood loss, length of stay and Ankle Osteoarthritis Scale (AOS) score 12 and 24 months after surgery were extracted [26] to compare the effectiveness of open arthrodesis and arthroscopic arthrodesis. These were the only available assessments of functional improvement that could be pooled from the studies.

**Table 1:** Characteristics of the included studies

Author	Year	Country	Journal	Lesion type	Procedure	Follow-up	Sample size	Arthroscopic	Open	Age	Male	BMI
										(A^a^ vs O^b^)	(A^a^ vs O^b^)	(A^a^ vs O^b^)
Meng [17]	2013	China	Chinese Journal of Reparative and Reconstructive Surgery	Ankle arthritis	Ankle fusion	12 months	30	14	16	Not available	Not available	Not available
O'Brien T S[18]	1999	USA	Foot Ankle International	Post-traumatic arthritis	Ankle fusion	NP	36	19	17	47.3 vs 44.6	9/19 vs 7/17	Not available
Nielsen KK [19]	2008	Denmark	Foot and Ankle Surgery	Post-traumatic arthritis	Ankle fusion	12 months	107	58	49	Not available	Not available	Not available
Townshed D [4]	2013	Canada	Journal of Bone and Joint Surgery Am	Post-traumatic/ primary OA	Ankle fusion	24 montsshs	60	30	30	59.4 vs 54.7	11/30 vs 20/30	27.4 vs 29.6
Myerson [20]	1990	USA	Clinical Orthopaedics and Related Research	Post-traumatic arthritis	Ankle fusion	23 months	33	17	16	Not available	7/17 vs 7/16	Not available
Peterson [21]	2010	USA	Journal of foot and Ankle Surgery	Ankle arthritis	Ankle fusion	6 months	10	10	10	56.2 vs 54.8	5/10 vs 6/10	37.36 vs 32.11
Panikkar [22]	2003	UK	Foot and Ankle Surgery	Osteoarthritis/rheumatoid arthritis/post-traumatic	Ankle fusion	96 months	41	21	22	65 vs 68	17/3 vs 12/9	
DeVrie s[23]	2019	USA	Journal of Foot and Ankle Surgery	Ankle instability	Ankle fusion	24.2 months	55	43	12	44.7 vs 39.5	16/43 vs 6/12	34.2 vs 33.1
Quayle [24]	2016	UK	Foot and Ankle Surgery	Post-traumatic arthritis	Ankle fusion	48 months	79	50	29	57 vs 61.9	37/50 vs 19/29	28.9 vs 28.0
Schmid [25]	2017	Canada	Foot Ankle International	End-stage ankle arthritis	Ankle fusion	54 months	97	62	35	57.4 vs 57.11	39/62 vs 26/35	28.2 v 28.8

^a^Arthroscopic arthordesis

^b^Open surgery

### Statistical analysis

For dichotomous variables (i.e., fusion rate, infection rate and overall complication rate), the odds ratios (OR) and weighted mean differences (WMD) were calculated and reported with 95% confidence intervals (CI) [27]. The algorithms proposed by Hozo et al. [18] were used when only the median, standard error or range was reported in the studies.

To assess statistical heterogeneity, a chi-square test with significance set at *p* < 0.10 was used, including in the meta-analysis, and I^2^ statistics quantified heterogeneity. A fixed-effect model was applied for all variables with I^2^ < 50%. This means that there was no significant heterogeneity among the studies. All meta-analyses were directed by Review Manager Version 5.3 (RevMan, Copenhagen: The Nordic Cochrane Center, The Cochrane Collaboration, 2014). A two-tailed test of significance (*p* < 0.05) was used.

The publication bias was assessed by the Stat (version 11) with the funnel plot and the Egger test. In order to check the stability of pooled outcomes, a sensitivity analysis was conducted. Both Begg’s test and Egger’s test were used for assessing publication bias. The statistical significance within all comparisons was mathematically signified as *p* < 0.05 [17].

## Results

### Study characteristics

Table 1 shows the characteristics of the 9 studies and their patients. Four of the 9 studies were conducted in the USA, 1 in Canada, 1 in China, 1 in Denmark and 1 in the UK. All the studies were published during the last 20 years, i.e., from 1990 to 2019. The studies included 3 prospective cohort studies and 7 retrospective cohort studies and enrolled a total of 507 patients with ankle arthritis. No randomised controlled trial was included. A total of 303 patients underwent arthroscopic arthrodesis, and 214 patients underwent open arthrodesis. In all cases, arthroscopy was used with standard anteromedial and anterolateral portals [25, 28].

**Fig. 2:**
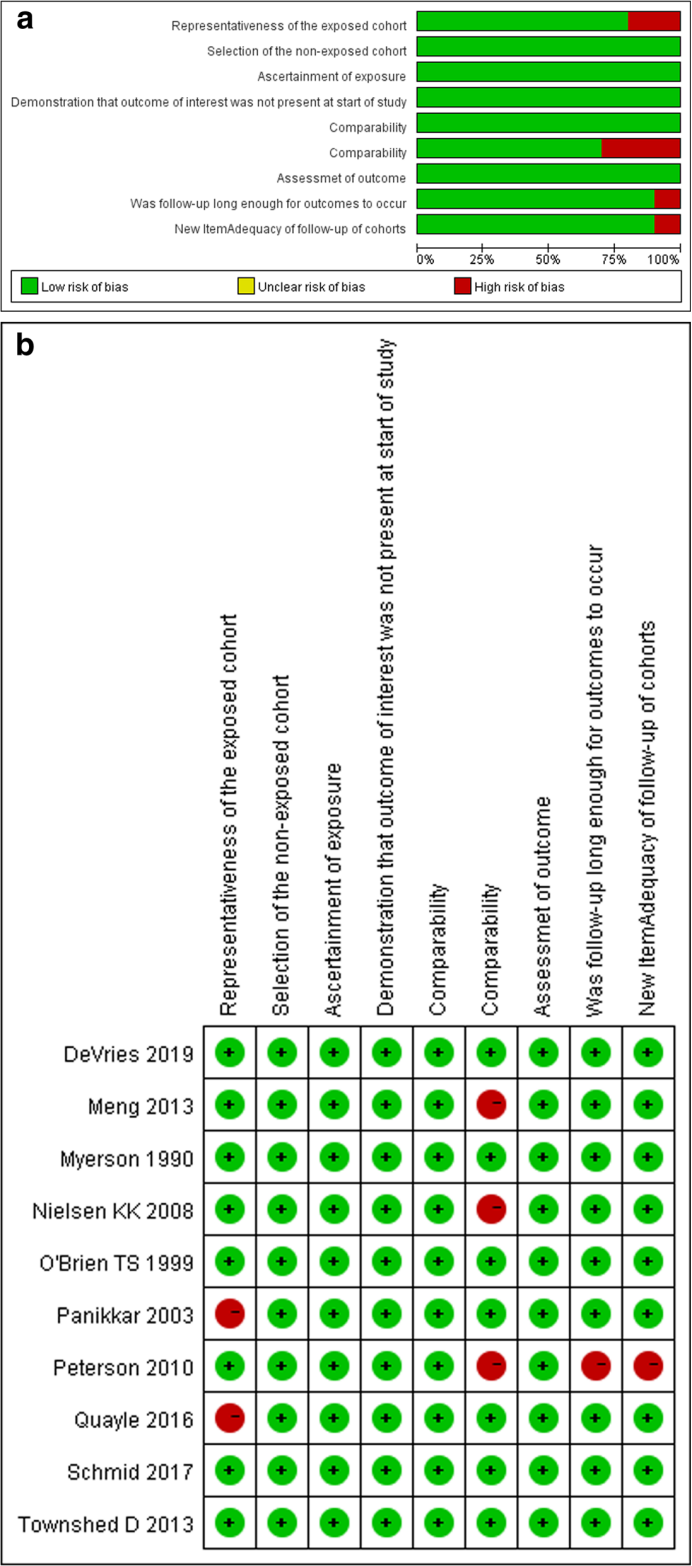
Graph of summary and results of the methodological quality assessment

Using the Newcastle–Ottawa scale, two analyses had a moderate risk of bias for study participation. One analysis had a high risk of bias. The graph in Fig. 2 demonstrates the summary and results of the methodological quality assessment. Regarding the MINORS, three articles had a score of 22. Five analyses had scores between 18 and 20. One article had a score of 16. Supplemental Table S1 contains a summary of the results**.**

Overall, methodologic limitations were examined in the studies with an average Coleman methodologic score (mean: 61; SD: 10.42; range: 43–75). The reasons with the low score were a short period of follow-up, high percentage coverage of retrospective studies and unclear procedure for assessing outcomes (Supplemental Table S2).

### Primary outcome

#### Fusion rate

**Fig. 3:**
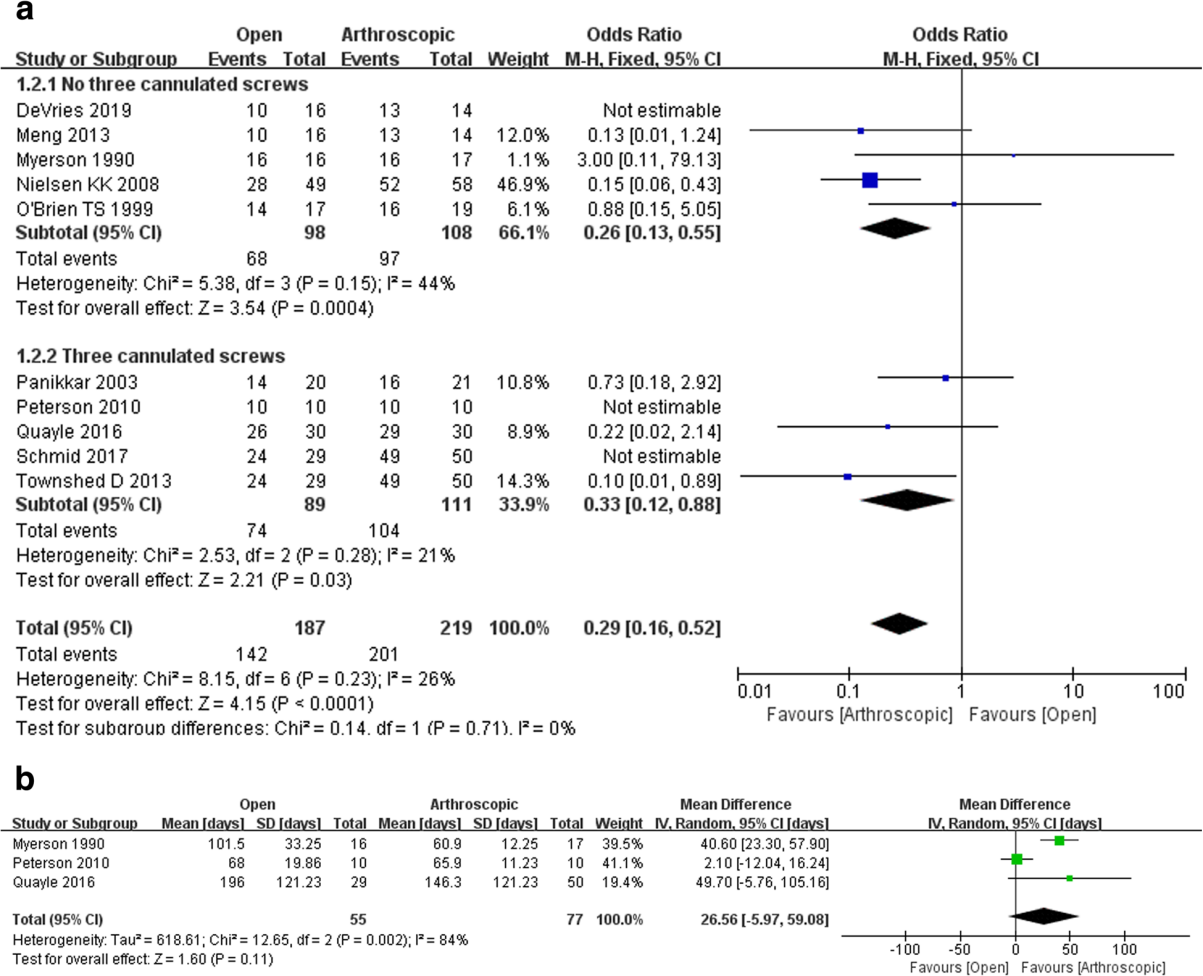
Primary outcomes on **a** fusion rate and **b** days to union

Seven studies reported the fusion rate, and the pooled data showed a significantly higher rate of fusion during arthroscopic arthrodesis compared with open surgery (odds ratio 0.25, 95% CI 0.11 to 0.57, *p* = 0.0001) in 128 of 167 patients. In addition, there was no significant heterogeneity between these two groups (for fusion rate assessing in arthroscopic arthrodesis with no three cannulated screws subgroup, 167 participants, odds ratio 0.26, 95% Cl 0.13 to 0.55, *p* = 0.0004; arthroscopic arthrodesis with three cannulated screws subgroup, 200 participants, odds ratio 0.33, 95% Cl 0.12 to 0.88, *p* = 0.03; Fig. 3a).

#### Days to union

Three studies assessed the days to union, and there was no difference between arthroscopic and open surgeries (WMD 1.62, 95% CI − 5.97 to 59.08, *p* = 0.11). There was no significant heterogeneity among these three studies (Fig. 3b).

### Surgical outcomes

#### Operation time

**Fig. 4:**
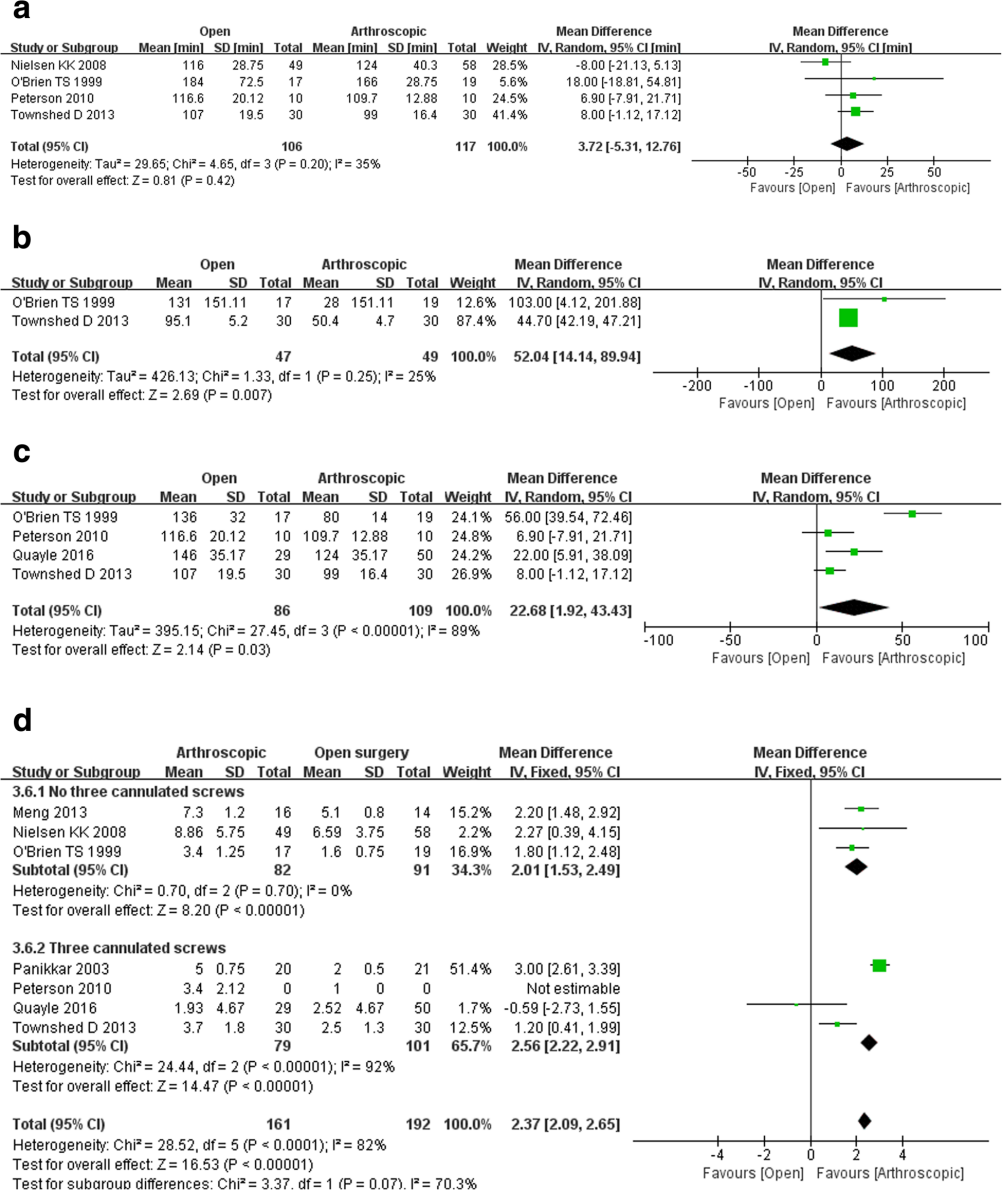
Surgical outcomes on **a** operation time, **b** estimated blood loss, **c** tourniquet time and **d** length of hospital stay

Four studies reported the operation time. The level of heterogeneity was low (chi-square = 4.65, df = 3, I^2^ = 35%, *p* = 0.20), and the pooled data from the four studies did not show a notable difference between arthroscopic arthrodesis and open surgery (WMD 3.72, 95% CI − 5.31, 12.76, *p* = 0.42; Fig. 4a).

#### Estimated blood loss

Two studies assessed the estimated blood loss during surgery, and the pooled data showed significantly less blood loss during arthroscopic arthrodesis compared with open surgery (WMD 52.04, 95% CI 14.14 to 89.94, *p* = 0.007). Also, there was no significant heterogeneity between the two groups (Fig. 4b).

#### Tourniquet time

Four studies reported the tourniquet time, and the pooled data showed remarkably shorter tourniquet times during arthroscopic arthrodesis compared with open surgery (WMD 22.68, 95% CI 1.92 to 43.43, *p* = 0.03). There was significant heterogeneity among these four studies (Fig. 4c).

#### Length of hospital stay

Six studies reported the length of stay in the hospital, and the pooled data showed markedly less hospitalisation time for patients undergoing arthroscopic arthrodesis compared with open surgery (WMD 1.62, 95% CI 0.97 to 2.26, *p* < 0.00001), with a low level of heterogeneity (chi-square = 8.21, df = 4, I^2^ = 51%, *p* = 0.08)—for length of hospital stay assessing in arthroscopic arthrodesis with no three cannulated screws subgroup, 173 participants, odds ratio 2.01, 95% Cl 1.53 to 2.49, *p* < 0.00001, while for that in arthroscopic arthrodesis with three cannulated screws subgroup, 180 participants, odds ratio 2.56, 95% Cl 2.22 to 2.91, *p* < 0.00001 (Fig. 4d).

### Complications

#### Overall complication rate

**Fig. 5:**
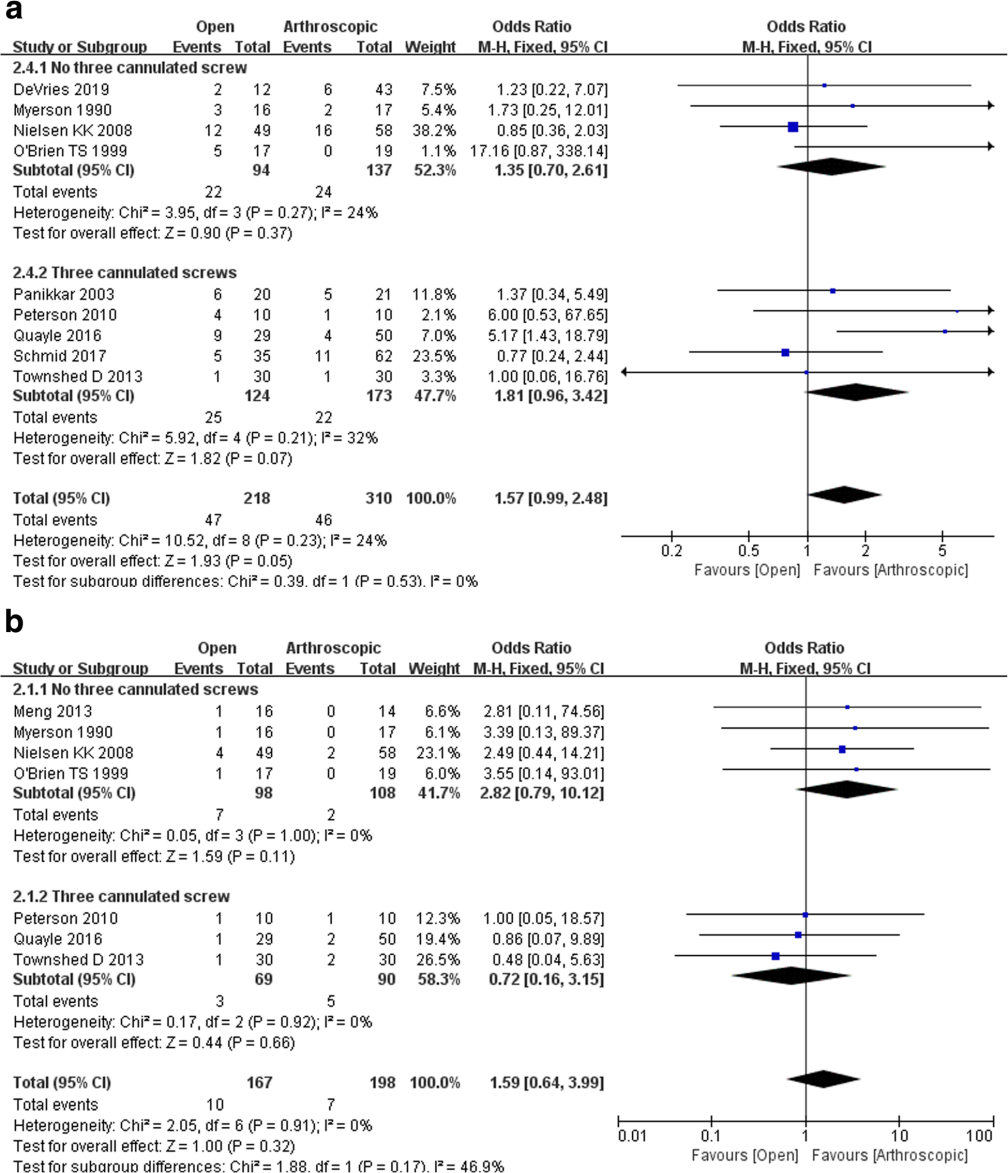
**a** Overall complications and **b** rate of infection

Eight studies assessed the overall complication rate, and there was no difference between arthroscopic arthrodesis and open surgery (WMD 1.70, 95% CI 0.84 to 3.43, *p* = 0.14). There was no obvious heterogeneity among these eight studies (for overall complication rate assessing in arthroscopic arthrodesis with no three cannulated screws subgroup, 231 participants, odds ratio 1.35, 95% Cl 0.70 to 2.61, *p* = 0.37; arthroscopic arthrodesis with three cannulated screws subgroup, 297 participants, odds ratio 1.81, 95% Cl 0.96 to 3.42, *p* = 0.07; Fig. 5a).

#### Rate of infection

Seven studies, including 365 patients, reported the rate of infection, and the pooled data showed no significant difference between patients who underwent arthroscopic arthrodesis and those who underwent open surgery (odds ratio 1.58, 95% CI 0.60 to 4.16, *p* = 0.36). There was no significant heterogeneity among these seven studies (for overall complication rate assessing in arthroscopic arthrodesis with no three cannulated screws subgroup, 206 participants, odds ratio 2.82, 95% Cl 0.79 to 10.12, *p* = 0.11; arthroscopic arthrodesis with three cannulated screws subgroup, 159 participants, odds ratio 0.72, 95% Cl 0.16 to 3.15, *p* = 0.66; Fig. 5b).

### Postoperative outcomes

#### One year post-surgery

**Fig. 6:**
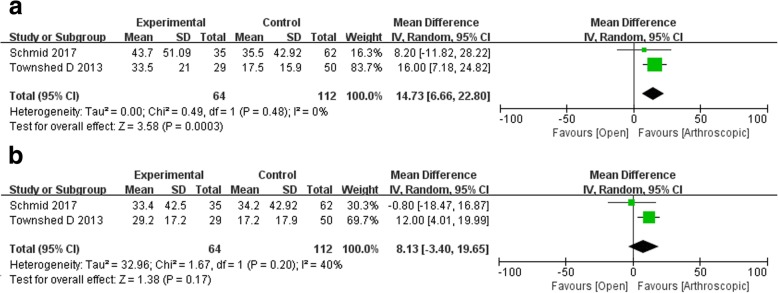
Postoperative outcomes **a** 1 year and **b** 2 years post-surgery

Two studies reported the 1-year postoperative recovery with the AOS score. The pooled data showed markedly better recovery for the patients who underwent arthroscopic arthrodesis compared with those who underwent open surgery (WMD 14.73, 95% CI 6.66 to 22.80, *p* = 0.0003) with a low heterogeneity (chi-square 0.49, df = 1, *p* = 0.48, I^2^ = 0%; Fig. 6a).

#### Two years post-surgery

Two studies assessed the 2-year postoperative recovery using the AOS scale and reported that patients who underwent arthroscopic arthrodesis had no notably greater recovery than those who underwent open surgery (WMD 8.13, 95% CI − 3.40 to 19.99, *p* = 0.48). In addition, there was no significant heterogeneity between these two groups (I^2^ = 40%, *p* = 0.20; Fig. 6b).

### Publication bias

**Fig. 7:**
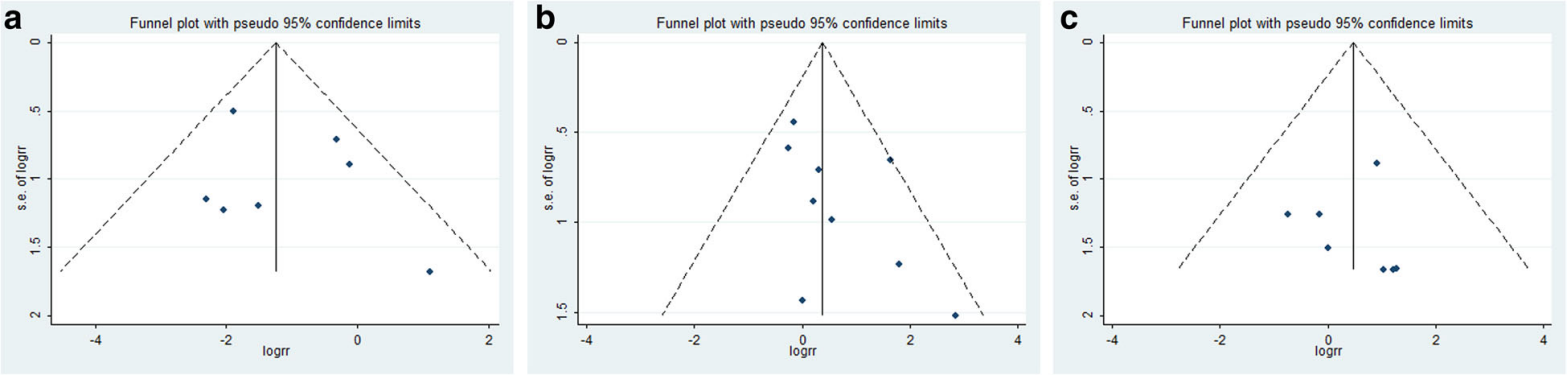
Funnel plots for the **a** fusion rate, **b** overall complication and **c** infection rate

Visual inspection of the Begg funnel plots for the fusion rate, overall complication and infection rate revealed symmetry (Fig. 7). To ensure that there was not publication bias, Egger’s test was also conducted (Supplemental Table S3). There was no statistically significant publication bias for any of the three results (95% Cl − 1.58–2.88, *p* = 0.75; 95% Cl − 0.96–2.97, *p* = 1.19; 95% Cl − 2.55–2.78, *p* = 0.92, respectively).

## Discussion

This study conducted a systematic review and meta-analysis of comparative studies published since 1990 to compare the fusion rate, operative effectiveness, safety and postoperative outcomes of arthroscopic arthrodesis and open surgery for ankle arthritis. Although there are similar studies comparing ankle arthrodesis and open surgery, this study included the most publications, reflecting the latest surgical results and focusing on the results of arthroscopic arthrodesis and open surgery for ankle arthritis. Considering limitations of a learning curve and insufficient evaluation, the study results might be meaningful.

### Strength of the systematic review

This study evaluated the two procedures using 4 perspectives: primary outcomes (fusion rate and days to union), surgical outcomes (operation time, estimated blood loss, tourniquet time and hospitalization length), complication outcomes (overall complication rate and infectious rate), and postoperative outcomes (AOS score at 12 and 24 months), unlike the previous study. These items formed a complete evaluation, from primary outcome to postoperative recovery. First, it was noted that there was no significant difference in days to union, which is contrary to previous studies [3]. Patients who underwent arthroscopic arthrodesis did not have a shorter time to union than those who experienced open surgery.

### Inferences and implications

Regarding the rate of fusion, the meta-analysis showed that there was a remarkable difference between arthroscopic arthrodesis and open surgery, and the data support the most recent meta-analysis by Honnenahalli et al. [3]. Patients who underwent arthroscopic arthrodesis had a higher fusion rate than those who underwent open surgery. In arthroscopic arthrodesis, the soft tissue envelope is disrupted to a minimum degree, which enables the major functions of soft tissues close to the surgical site. The bone healing cascade is activated rapidly, so the bone heals rapidly and function improves in the early stage due to the minimum degree of soft-tissue envelope disruption [4, 19]. The limited exposure causes significantly decreased periosteal stripping [20]. These theories may elucidate the high fusion rate for arthroscopic arthrodesis.

Additionally, this study is the first to assess the estimated blood loss, and the pooled data significantly favoured arthroscopic arthrodesis compared with open surgery. The data were pooled by O’Brien et al. [21] and Townshed et al. [4]. Moreover, the tourniquet time during the operation was considerably shorter with arthroscopic arthrodesis than with open surgery. Although Meng et al. [23] mentioned that the operation time of the arthroscopic group was significantly longer than that of the open group, this study showed no significant differences in the operating time for the two procedures. There is only one relevant study mentioned in Meng et al. [23], but their pooled data were extracted from a larger sample size from different countries. Therefore, the risk of bias is minimised in the results. As a result, arthroscopic arthrodesis does not take longer to complete than open surgery.

Regarding complications, patients who undergo arthroscopic arthrodesis may require removal of a screw for prominence, superficial infections, deep vein thromboses/pulmonary emboli, fixation revision, stress fracture or deep infections after surgery [24]. The most common complications were non-union, delayed union and infection [28]. However, the study shows no significant difference between these two surgical procedures. It has been reported that patients require reoperation for similar complications in the two groups. This explains why there was no remarkable difference in either group given the similar postoperative radiological alignment [22]. The infection rates for the open surgery group and the arthroscopic group did not differ significantly. Some reports have shown results with infection rates in the open surgery group from 0 to 9% and very low infection rates in the arthroscopic technique group [29].

Moreover, postoperative improvements were studied via the AOS score. The arthroscopic arthrodesis group showed significantly better scores at 1 year compared with the open surgery groups. However, no significant difference between the groups at 2 years was noted. Since less area was damaged during arthroscopic arthrodesis than during open surgery, the tissues and functions recovered rapidly and in earlier stages, as per Townshend et al. [4]. Further study is needed to improve understanding of the clinical picture of the finding, especially with regards to education for patients choosing the best time to undergo reconstruction for their ankle arthritis [22]. This is a new finding compared with the previous study. Thus, patients who underwent arthroscopic arthrodesis recovered in a shorter time but showed similar bone reconstruction in the long term compared with those who underwent open surgery.

### Limitation of the systematic review

There are several limitations despite these findings. First, the quality of the primary studies valued the final quality of the meta-analysis. Ten level VI studies were included. The inferences from this study may be limited. Second, a larger sample size is needed for some of the results, such as blood loss and functional improvement, which are drawn from few data points because they were not investigated in all the studies that were available for review. Most of our findings are similar with previous study that found very limited evidence of any benefit of open and arthroscopic fusion. Third, more longitudinal studies are needed since a 24-month follow-up cannot show the long-term effects or complications of the procedures [30].

Overall, we have assessed the comparison between arthroscopic arthrodesis and open surgery. To date, the fusion rate, tourniquet time and hospitalisation length have demonstrated the benefit of arthroscopic arthrodesis. According to Ling et al. [8], the effects of ankle arthrodesis on biomechanics or whether ankle arthrodesis leads to adjacent-joint arthritis are still uncertain without any common consensus. More benefit claims should be proved by data from well-designed trials in the future. Future researchers investigating any related treatment for end-stage arthritis should provide a common set of outcomes, and the outcome trials should be standardised.

## Conclusion

Arthroscopic arthrodesis was associated with a higher fusion rate, shorter tourniquet time and shorter length of hospitalisation than open surgery. Nevertheless, we need to interpret the results with caution, pool RCT studies with larger sample sizes and perform comparative studies which will be required in prospective studies to evaluate the efficiency of arthroscopic arthrodesis.

## Supplementary information

**Additional file 1: Table S1.** Methodological Index for Non-randomized Studies (MINORS) Assessment.

**Additional file 2: Table S2.** Modified Coleman methodology.

**Additional file 3: Table S3.** Published bias

**Additional file 4: Table S4.** The Difference of Arthroscopic Arthrodesis.

## Data Availability

The authors confirm that the data supporting the findings of this study are available within the article and its supplementary materials.

## Abbreviations

AOS: Ankle Osteoarthritis Scale
BMI: Body mass index
CI: Confidence intervals
MINORS: Methodological index for non-randomised studies
NOS: Newcastle–Ottawa Scale
OA: Osteoarthritis
OR: Odds ratios
SD: Standard deviation
WMD: Weighted mean differences

## References

[1] Ewalefo Samuel O., Dombrowski Malcolm, Hirase Takashi, Rocha Jorge L., Weaver Mitchell, Kline Alex, Carney Dwayne, Hogan MaCalus V. (2018). Management of Posttraumatic Ankle Arthritis: Literature Review. Current Reviews in Musculoskeletal Medicine.

[2] Glyn-Jones S (2015). Osteoarthritis. Lancet.

[3] Honnenahalli Chandrappa M, Hajibandeh S, Hajibandeh S (2017). Ankle arthrodesis-open versus arthroscopic: a systematic review and meta-analysis. J Clin Orthopaedics Trauma.

[4] Townshend D (2013). Arthroscopic versus open ankle arthrodesis: a multicenter comparative case series. J Bone Joint Surg Am.

[5] Mendicino SS, Kreplick AL, Walters JL (2017). Open ankle arthrodesis. Clin Podiatr Med Surg.

[6] Wang Y (2015). Effects of ankle arthrodesis on biomechanical performance of the entire foot. PLoS One.

[7] Kim HJ (2017). Total ankle arthroplasty versus ankle arthrodesis for the treatment of end-stage ankle arthritis: a meta-analysis of comparative studies. Int Orthop.

[8] Ling J (2015). Investigating the relationship between ankle arthrodesis and adjacent-joint arthritis in the hindfoot: a systematic review. J Bone Joint Surg Am.

[9] Piraino JA, Lee MS (2017). Arthroscopic ankle arthrodesis: an update. Clin Podiatr Med Surg.

[10] Elmlund AO, Winson IG (2015). Arthroscopic ankle arthrodesis. Foot Ankle Clin.

[11] Wagner E, Melo R (2018). Subtalar arthroscopic fusion. Foot Ankle Clin.

[12] Gharehdaghi M, Rahimi H, Mousavian A (2014). Anterior ankle arthrodesis with molded plate: technique and outcomes. Arch Bone Jt Surg.

[13] Lo CK-L, Mertz D, Loeb M (2014). Newcastle-Ottawa scale: comparing reviewers' to authors' assessments. BMC Med Res Methodol.

[14] Pinski JM (2016). Low level of evidence and methodologic quality of clinical outcome studies on cartilage repair of the ankle. Arthroscopy.

[15] Sobieraj DM (2018). Association of inhaled corticosteroids and long-acting β-agonists as controller and quick relief therapy with exacerbations and symptom control in persistent asthma: a systematic review and meta-analysis. Jama.

[16] Peterson KS, Lee MS, Buddecke DE (2010). Arthroscopic versus open ankle arthrodesis: a retrospective cost analysis. J Foot Ankle Surg.

[17] Wang Y (2012). Auditing complex concepts of SNOMED using a refined hierarchical abstraction network. J Biomed Inform.

[18] Wang S (2020). Efficacy of totally laparoscopic compared with laparoscopic-assisted total gastrectomy for gastric cancer: a meta-analysis. World J Clin Cases.

[19] Szczęsny Grzegorz (2018). Fracture Repair: Its Pathomechanism and Disturbances. Trauma Surgery.

[20] Myerson, M.S. and G. Quill, Ankle arthrodesis. A comparison of an arthroscopic and an open method of treatment. Clinical orthopaedics and related research, 1991(268): p. 84-95. 2060232

[21] O'Brien TS (1999). Open versus arthroscopic ankle arthrodesis: a comparative study. Foot Ankle Int.

[22] Schmid T (2017). Effect of preoperative deformity on arthroscopic and open ankle fusion outcomes. Foot Ankle Int.

[23] Meng Q (2013). Effectiveness comparison between arthroscopic and open ankle arthrodeses. Zhongguo Xiu Fu Chong Jian Wai Ke Za Zhi.

[24] Ovaska M (2015). Complications in ankle fracture surgery. Acta Orthopaedica.

[25] DeVries JG, Scharer BM, Romdenne TA (2019). Ankle stabilization with arthroscopic versus open with suture tape augmentation techniques. J Foot Ankle Surg.

[26] Croft S (2017). Association of ankle arthritis score with need for revision surgery. Foot Ankle Int.

[27] Nuzzo Regina L. (2019). Making Continuous Measurements into Dichotomous Variables. PM&R.

[28] Quayle J (2018). Arthroscopic versus open ankle arthrodesis. Foot and ankle surgery.

[29] Nielsen KK, Linde F, Jensen NC (2008). The outcome of arthroscopic and open surgery ankle arthrodesis: a comparative retrospective study on 107 patients. Foot and ankle surgery.

[30] Panikkar KV. A comparison of open and arthroscopic ankle fusion. Foot and Ankle Surgery. 2003.

