# Supplementary material for: Open versus arthroscopic ankle arthrodesis: a systematic review and meta-analysis
Source: J Orthop Surg Res. 2020 May 24;15:187. doi: 10.1186/s13018-020-01708-4 (PMC7247192; doi:10.1186/s13018-020-01708-4)
Supplement: Supplementary file 1 — Additional file 1: Table S1. Methodological Index for Non-randomized Studies (MINORS) Assessment. [file 13018_2020_1708_MOESM1_ESM.docx]

|  | **Clearly stated aim** | **Inclusion of consecutive patients** | **Prospective data collection** | **Endpoints appropriate to study aim** | **Follow-up period appropriate to study aim** | **<5% lost to follow-up** | **Prospective calculation of study size** | **Adequate control group** | **Contemporary groups** | **Baseline equivalence of groups** | **Adequate statistical analyses** | **Total** |
| --- | --- | --- | --- | --- | --- | --- | --- | --- | --- | --- | --- | --- |
| **DeVries^21^** | 2 | 2 | 2 | 2 | 2 | 2 | 2 | 2 | 2 | 2 | 2 | 22/24 |
| **Meng^26^** | 2 | 2 | 2 | 2 | 2 | 0 | 2 | 0 | 0 | 2 | 2 | 16/24 |
| **Myerson^24^** | 2 | 2 | 2 | 2 | 2 | 0 | 1 | 1 | 2 | 2 | 2 | 18/24 |
| **Nielsen KK^29^** | 2 | 2 | 2 | 2 | 2 | 0 | 2 | 2 | 2 | 2 | 2 | 22/24 |
| **O'Brien TS^25^** | 2 | 2 | 2 | 2 | 1 | 0 | 2 | 2 | 2 | 2 | 2 | 19/24 |
| **Peterson^16^** | 2 | 2 | 2 | 2 | 1 | 0 | 2 | 2 | 2 | 2 | 2 | 19/24 |
| **Panikkar^30^** | 2 | 2 | 2 | 2 | 2 | 2 | 2 | 2 | 2 | 2 | 2 | 24/24 |
| **Quayle^22^** | 2 | 1 | 2 | 2 | 2 | 0 | 1 | 2 | 2 | 2 | 2 | 18/24 |
| **Schmid^28^** | 2 | 2 | 2 | 2 | 2 | 0 | 2 | 2 | 2 | 2 | 2 | 20/24 |
| **Townshed^4^** | 2 | 2 | 2 | 2 | 2 | 2 | 2 | 2 | 2 | 2 | 2 | 22/24 |

**TABLE 2**

Methodological Index for Non-randomized Studies (MINORS) Assessment
