# Supplementary material for: Open versus arthroscopic ankle arthrodesis: a systematic review and meta-analysis
Source: J Orthop Surg Res. 2020 May 24;15:187. doi: 10.1186/s13018-020-01708-4 (PMC7247192; doi:10.1186/s13018-020-01708-4)
Supplement: Supplementary file 2 — Additional file 2: Table S2. Modified Coleman methodology. [file 13018_2020_1708_MOESM2_ESM.docx]

|  | Max CMS | Mean | SD | Variance, σ^2^ | Range |
| --- | --- | --- | --- | --- | --- |
| Part A | 60 | 33 | 8.32 | 69.2 | 22-46 |
| 1. Study size | 10 | 5 | 3.06 | 9.4 | 0-10 |
| 1. Follow up | 5 | 1.7 | 1.42 | 2.01 | 0-5 |
| 1. Surgical procedures (n) | 10 | 7 | 0 | 0 | 7 |
| 1. Type of study | 15 | 4 | 4.9 | 24 | 0-10 |
| 1. Diagnostic certainty | 5 | 5 | 0 | 0 | 5 |
| 1. Description of surgical technique | 5 | 4.8 | 0.6 | 0.36 | 3-5 |
| 1. Description of post-op rehabilition | 10 | 5.5 | 2.69 | 7.25 | 0-10 |
| Part B | 50 | 28 | 2.72 | 7.4 | 21-31 |
| 1. Outcome criteria | 10 | 8.7 | 1.1 | 1.21 | 7-10 |
| 1. Procedure for assessing outcome | 15 | 6.8 | 1.6 | 2.56 | 6-10 |
| 1. Description of subject selection process | 15 | 12.5 | 1.5 | 2.25 | 8-13 |
| Coleman Methodology Score (parts A+B) | 100 | 61 | 10.42 | 108.6 | 43-75 |

The coleman methodology score

Table shows the overall Coleman Methodology Score of the 10 included studies.
