# Supplementary material for: Open versus arthroscopic ankle arthrodesis: a systematic review and meta-analysis
Source: J Orthop Surg Res. 2020 May 24;15:187. doi: 10.1186/s13018-020-01708-4 (PMC7247192; doi:10.1186/s13018-020-01708-4)
Supplement: Supplementary file 3 — Additional file 3: Table S3. Published bias [file 13018_2020_1708_MOESM3_ESM.docx]

**Supplemental Table 2**

Egger test for publication bias

i

| Std_Eff | Coef. | Std.Err. | t | P> \|t\| | [95% Conf. | Interval] |
| --- | --- | --- | --- | --- | --- | --- |
| slop | -1.973405 | 1.050249 | -1.88 | 0.119 | -4.673156 | 0.726347 |
| bias | 0.9166624 | 1.234153 | 0.74 | 0.491 | -2.255828 | 4.089153 |

ii

| Std_Eff | Coef. | Std.Err. | t | P> \|t\| | [95% Conf. | Interval] |
| --- | --- | --- | --- | --- | --- | --- |
| slop | -0.1344613 | 0.5338709 | -0.25 | 0.808 | -1.396865 | 1.127943 |
| bias | 0.9960557 | 0.8336117 | 1.19 | 0.271 | -0.9751227 | 2.967234 |

iii

| Std_Eff | Coef. | Std.Err. | t | P> \|t\| | [95% Conf. | Interval] |
| --- | --- | --- | --- | --- | --- | --- |
| slop | -0.7488902 | 0.6735464 | -1.11 | 0.303 | -2.341574 | 0.843794 |
| bias | 1.624538 | 0.9027078 | 1.80 | 0.115 | -0.5100269 | 3.759103 |

*i: Published bias for fusion rate

ii: Published bias for overall complication

iii: Published bias for infectious rate
