# Supplementary material for: Open versus arthroscopic ankle arthrodesis: a systematic review and meta-analysis
Source: J Orthop Surg Res. 2020 May 24;15:187. doi: 10.1186/s13018-020-01708-4 (PMC7247192; doi:10.1186/s13018-020-01708-4)
Supplement: Supplementary file 4 — Additional file 4: Table S4. The Difference of Arthroscopic Arthrodesis. [file 13018_2020_1708_MOESM4_ESM.docx]

|  |  | Surgical Approach | |  | Fixation | | | | |
| --- | --- | --- | --- | --- | --- | --- | --- | --- | --- |
|  | Surgeon | Anteromedial portal | Anterolateral portal |  | Two cannulated screws | Three cannulated screws | Four screw technique | Compression screw fixation | Non-invasive distractor |
| **DeVries^21^** | J.G.D. and B.M.S. | ✓ | ✓ |  | 🗶 | 🗶 | 🗶 | 🗶 | ✓ |
| **Meng^26^** | N/A | ✓ | ✓ |  | 🗶 | 🗶 | 🗶 | ✓ | 🗶 |
| **Myerson^24^** | N/A | ✓ | ✓ |  | 🗶 | 🗶 | 🗶 | 🗶 | ✓ |
| **Nielsen KK^29^** | N/A | ✓ | ✓ |  | ✓ | 🗶 | 🗶 | 🗶 | 🗶 |
| **O'Brien TS^25^** | one surgeon at the Medical College of Wisconsin | ✓ | ✓ |  | ✓ | 🗶 | 🗶 | 🗶 | ✓ |
| **Peterson^16^** | N/A | ✓ | ✓ |  | ✓ | ✓ | 🗶 | 🗶 | 🗶 |
| **Panikkar^30^** | D.E.B. and M.S.L. | ✓ | ✓ |  | ✓ | ✓ | 🗶 | 🗶 | 🗶 |
| **Quayle^22^** | Five fellowship trained foot and ankle surgeons | ✓ | ✓ |  | ✓ | ✓ | ✓ | ✓ | ✓ |
| **Schmid^28^** | Two surgeons | ✓ | ✓ |  | ✓ | ✓ | 🗶 | 🗶 | ✓ |
| **Townshed^4^** | Three orthopedic surgeons | ✓ | ✓ |  | ✓ | ✓ | 🗶 | 🗶 | ✓ |

**The Difference of the Arthroscopic Arthrodesis**
